# Flip-Flop Pharmacokinetics in Pregnancy and the Future of Obstetric Pharmacology

**DOI:** 10.1002/jcph.70229

**Published:** 2026-06

**Authors:** Ahizechukwu C. Eke

**Affiliations:** Division of Maternal Fetal Medicine & Clinical Pharmacology, Department of Gynaecology and Obstetrics, Johns Hopkins University School of Medicine, Baltimore, MD, USA

**Keywords:** absorption-limited kinetics, biologics, flip-flop pharmacokinetics, long-acting injectables, model-informed drug development, pregnancy

## Introduction

Pregnancy fundamentally alters the pharmacologic landscape through dynamic changes in gastrointestinal physiology, tissue perfusion, plasma volume, adipose distribution, protein binding, renal clearance, placental transport, and immunologic regulation.^1–3^ Most pregnancy pharmacokinetic discussions have therefore centered on altered clearance and volume of distribution. This emphasis is appropriate for many small molecules, but is incomplete for modern therapeutic platforms whose systemic exposure is governed by slow release from an absorption site.^4^ Long-acting injectables, subcutaneous biologics, implantable systems, depot formulations, and extended-release formulations increasingly place absorption at the center of the concentration–time profile.^4^

*Flip-flop pharmacokinetics* occurs when the rate of drug absorption becomes slower than the rate of elimination.^5,6^ Under this condition, the terminal slope of the plasma concentration–time curve reflects continued absorption kinetics rather than elimination kinetics alone.^5^ This distinction is not merely semantic. A drug may appear to have a long half-life because it is slowly entering the circulation from a depot, subcutaneous space, formulation matrix, tissue reservoir, or biologic transport pathway, even when systemic elimination remains efficient. In pregnancy, this issue becomes clinically relevant because the physiologic determinants of absorption and tissue release change across gestation and reverse postpartum.^2^ Thus, direct pregnancy-specific evidence for true flip-flop kinetics remains limited for many agents.

However, the convergence of long-acting therapeutics with gestational changes in gastrointestinal transit, regional perfusion, extracellular fluid expansion, adipose remodeling, lymphatic uptake, maternal metabolism, placental transport, and biologic disposition creates a plausible and increasingly important framework for pregnancy pharmacology. The goal is, therefore, not to declare flip-flop kinetics ubiquitous in pregnancy, but to highlight when it should be prospectively considered, measured, modeled, and distinguished from altered elimination.

## The General Pharmacologic Concept of Flip-Flop Pharmacokinetics

Under conventional first-order extravascular pharmacokinetics, absorption occurs more rapidly than elimination.^1,2^ In a simplified one-compartment oral model, the absorption rate constant is greater than the elimination rate constant. Stated conceptually:

${\text{K}}_{\text{a}}\;>\;{\text{K}}_{\text{e}}$where ${\text{K}}_{\text{a}}$ represents the absorption rate constant, and ${\text{K}}_{\text{e}}$ represents the elimination rate constant. Consequently, the terminal elimination phase of the concentration–time curve primarily reflects systemic elimination processes, and the observed half-life approximates elimination half-life.

In contrast, flip-flop pharmacokinetics occurs when absorption becomes slower than elimination:

$${\text{K}}_{\text{a}}\;<\;{\text{K}}_{\text{e}}$$

Under flip-flop conditions, absorption becomes the slower process, so the terminal slope reflects the rate at which drug continues to enter the systemic circulation.^5,6^ In this setting, apparent half-life therefore becomes absorption-limited rather than elimination-limited.

The mathematics are simple in principle because the terminal slope of a standard first-order extravascular profile is governed by the slower of the two rate constants. The interpretive problem is not the algebra but the identifiability. In a one-compartment model fitted only to extravascular concentration-time data, ${\text{K}}_{\text{a}}$ and ${\text{K}}_{\text{e}}$ can be difficult to distinguish unless there is informative early and late sampling, an intravenous reference phase, formulation comparisons, or mechanistic knowledge.^5^ Therefore, a long terminal half-life after an oral, intramuscular, or subcutaneous dose can be incorrectly attributed to slow elimination when it actually reflects slow absorption—Table 1.^7^ This is the central identification problem that makes flip-flop kinetics important for clinical interpretation and drug development.

Flip-flop kinetics is a general phenomenon and is not specific to pregnancy. Its relevance in pregnancy arises because pregnancy can change both sides of the rate–constant relationship.^1^ Pregnancy modifies the drug absorption process through altered gastrointestinal transit, tissue perfusion, interstitial fluid, adipose architecture, lymphatic transport, and depot dissolution.^1–3,8^ Pregnancy also modifies drug elimination through changes in drug clearance, hepatic enzyme activity, transporter expression, protein binding, and volume of distribution.^1–3,8^ Pregnancy can therefore shift a drug toward or away from absorption-limited behavior depending on the relative magnitude and direction of changes in absorption and elimination.

## Why Pregnancy Creates Conditions Favorable for Flip-Flop Kinetics—Pregnancy Physiology and Absorption-Limited Exposure

Pregnancy introduces multiple physiologic changes capable of altering absorption kinetics across oral, intramuscular, subcutaneous, transdermal, and biologic therapeutic platforms^1,3,8,9^ (Table 1).

### Gastrointestinal Physiology

Pregnancy-associated progesterone elevation slows gastric emptying and intestinal motility.^1,3,8,9^ Delayed gastrointestinal transit prolongs dissolution and intestinal absorption phases for orally administered extended-release formulations.^1–3^ While these changes do not necessarily produce flip-flop kinetics when absorption remains limited for conventional immediate-release drugs under these conditions, they become more relevant for extended-release formulations, drugs with dissolution-limited absorption, or products whose absorption phase is already prolonged when used in pregnancy.

### Altered Tissue Perfusion and Depot Dynamics

Intramuscular and subcutaneous drugs such as long-acting injectables depend heavily on local tissue perfusion, depot dissolution, lymphatic uptake, and regional vascular absorption.^4–6^ Pregnancy alters skeletal muscle blood flow, adipose composition, extracellular fluid volume, and interstitial pressure gradients.^1,2,8,9^ As such, gestational weight gain, adipose expansion, pregnancy-related edema, and changes in interstitial pressure modify the microenvironment surrounding depot injections.^1,2,8,9^ These changes alter release kinetics from intramuscular or subcutaneous depots. Long-acting cabotegravir and rilpivirine provide clinically important contemporary examples. These formulations rely on prolonged release from injection depots over weeks to months.^10,11^ In pregnancy, altered tissue physiology can modify depot dissolution and systemic absorption kinetics, potentially prolonging or attenuating exposure trajectories.^4^

### Increased Volume of Distribution

Pregnancy-associated increases in total body water and adipose tissue alter drug partitioning into peripheral compartments.^1,2^ Lipophilic agents demonstrate prolonged redistribution kinetics, further contributing to delayed systemic equilibration and longer terminal phases.^1–3^ The contribution of volume of distribution should be interpreted cautiously because it is not a direct cause of flip-flop kinetics. Rather, its effect is mediated through the elimination rate constant $\left({\text{K}}_{\text{e}}\right)$:

$${\text{K}}_{\text{e}}=\text{Clearance/Volume of distribution}.$$

**Figure 1.**
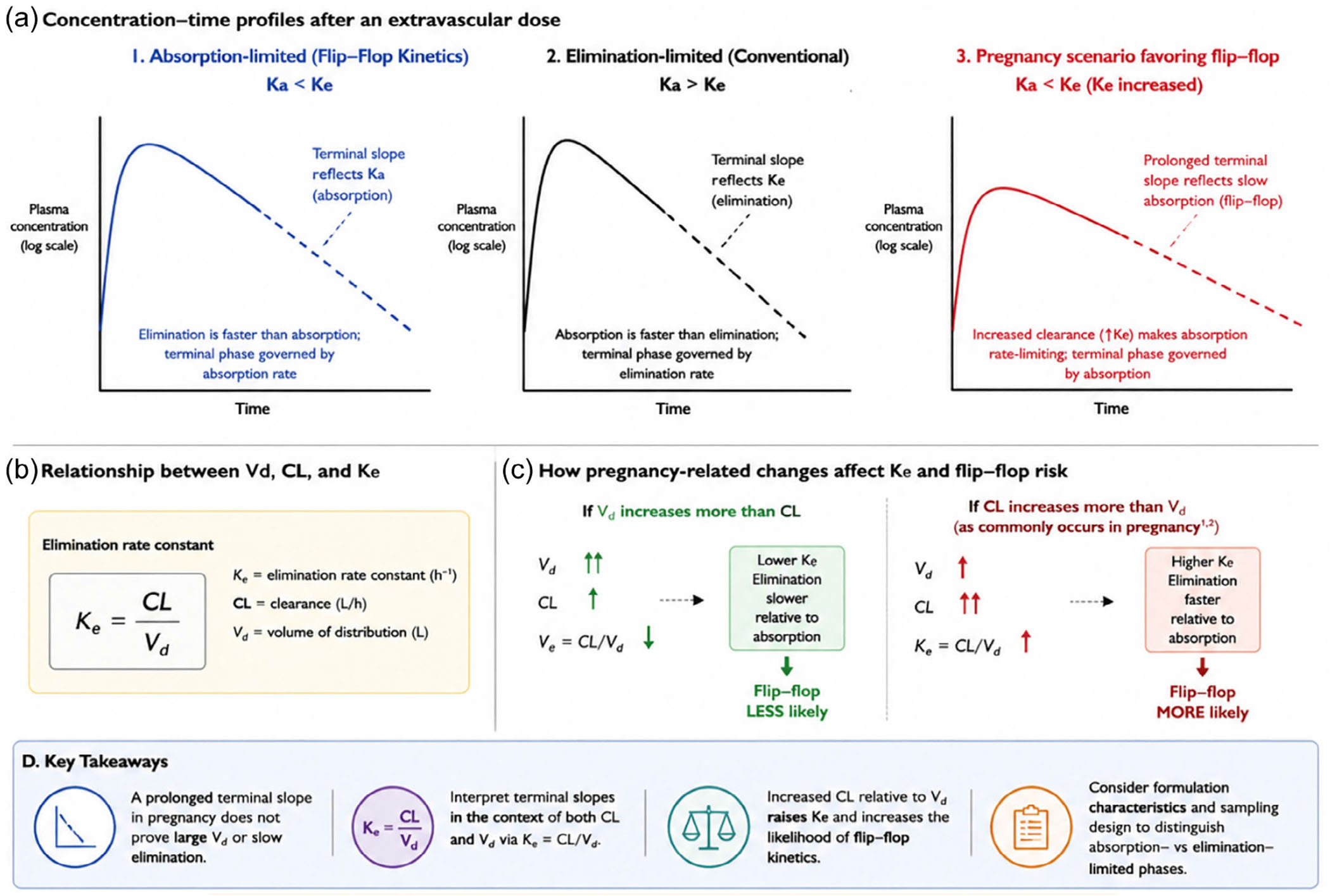
Flip-flop pharmacokinetics and the role of clearance and volume of distribution in pregnancy.

Rowland and Tozer describe that the elimination rate constant $\left({\text{K}}_{\text{e}}\right)$ is determined by the ratio of clearance to volume of distribution $\left({\text{K}}_{\text{e}}{=\text{CL}/\text{V}}_{\text{d}}\right)$.^12^ Thus, changes in terminal slopes must be interpreted in the context of both parameters rather than volume of distribution alone. If volume of distribution increases disproportionately to clearance, ${\text{K}}_{\text{e}}$ decreases, making absorption-limited elimination less likely. Conversely, when clearance increases more than volume of distribution, as is common in pregnancy,^1,2^ ${\text{K}}_{\text{e}}$ increases and absorption is more likely to become rate-limiting (Figure 1). Therefore, the impact of pregnancy on terminal concentration–time profiles should be considered in the context of both clearance and volume of distribution, together with formulation characteristics and sampling design, rather than attributing prolonged terminal slopes to volume of distribution alone.

### Maternal and Placental Metabolism

Pregnancy induces or suppresses specific metabolic pathways, including changes in CYP3A, CYP2D6, CYP2C9, CYP1A2, uridine diphosphate glucuronosyltransferase activity, renal transport, and efflux transporters.^1,2,8,9^ The placenta contributes additional transport and metabolic functions through P-glycoprotein, breast cancer resistance protein, organic anion, and cation transporters, and enzymes expressed in trophoblast and placental tissues.^1,2^ These pathways influence maternal–fetal distribution, fetal exposure, and concentration gradients that interact with prolonged absorption.

### Protein Binding and Monoclonal Antibody Disposition

Biologics and monoclonal antibodies introduce additional complexity. Unlike small molecules, biologics are not primarily metabolized through hepatic cytochrome (CYP) P450 pathways. Instead, they undergo cellular uptake, FcRn-mediated recycling, lysosomal degradation, and target-mediated clearance.^2^ Pregnancy alters Fc receptor expression, placental IgG transport, immunoglobulin turnover, inflammatory signaling, and plasma protein concentrations.^13,14^ These changes influence both systemic exposure and tissue redistribution kinetics. Importantly, prolonged biologic exposure may reflect sustained tissue release and receptor recycling rather than conventional elimination.

## Long-Acting Therapeutics and the Emergence of Pregnancy-Specific Flip-Flop Systems

The increasing use of long-acting therapeutics in reproductive-aged populations has transformed the practical relevance of flip-flop pharmacokinetics (Table 2).

### Long-Acting Antiretrovirals

Long-acting antiretrovirals illustrate why this framework matters. Injectable cabotegravir and rilpivirine represent particularly important examples because reproductive-aged women are major end-users of HIV prevention and treatment strategies.^10,11^ Both drugs are designed to maintain concentrations for weeks to months after intramuscular injection. Their prolonged pharmacokinetic “tail” primarily reflects sustained release from the injection depot and continued systemic entry rather than delayed elimination alone. In pregnancy, direct data remain sparse, but a recent case-series measuring long-acting cabotegravir and rilpivirine during the second and third trimesters reported maintained concentrations above proposed efficacy thresholds with monthly dosing, whereas every 2-month dosing produced sub-therapeutic rilpivirine concentrations in the reported patients.^11^ These findings do not prove pregnancy-specific flip-flop kinetics, but they demonstrate why pregnancy studies of long-acting agents must characterize the absorption phase, the tail phase, and postpartum transitions explicitly.

### Depot Hormonal Therapies

Depot hormonal therapies provide another relevant example. Depot medroxyprogesterone acetate and 17-hydroxyprogesterone caproate are administered at intervals that inherently depend on prolonged release from tissue reservoirs.^15^ In such systems, the observed terminal profile is shaped substantially by depot release and formulation properties. Pregnancy-specific pharmacokinetic studies of hydroxyprogesterone caproate have demonstrated wide variability in exposure, reinforcing that depot-based therapeutics cannot be understood solely through systemic clearance.

### Biologics and Monoclonal Antibodies

Subcutaneous biologics and monoclonal antibodies add a mechanistic layer that differs from small molecules. Once absorbed, monoclonal antibodies undergo cellular uptake, neonatal Fc receptor recycling, lysosomal degradation, target-mediated clearance, and transplacental FcRn-mediated transfer.^13,14^ Pregnancy changes immunoglobulin concentrations, inflammatory state, placental FcRn-mediated transport, and maternal protein binding, altering both maternal and fetal exposure.^2^ These factors can alter both maternal and fetal exposure. For biologics, prolonged terminal profiles reflect continued lymphatic entry, receptor recycling, target binding, or tissue re-distribution rather than classic hepatic or renal elimination.

## Methodologic Implications of Flip-Flop Pharmacokinetics for Pregnancy Pharmacology

Recognition of flip-flop kinetics changes how pregnancy pharmacokinetics should be designed and interpreted. Dosing intervals and washout periods are ultimately driven by exposure and pharmacodynamic risk, not by terminology. However, accurate prediction of exposure, persistence, and subtherapeutic “tail” phases depend on knowing whether the terminal slope reflects elimination or continued absorption.^5^ If the apparent half-life is absorption-limited, then a washout interval on presumed elimination half-life may be misleading in pregnancy. For long-acting antiretrovirals, this distinction is clinically relevant because low concentrations after discontinuation can create a resistance risk.^10,11^ For biologics, it is relevant because maternal and fetal exposure can persist through receptor recycling and placental transfer even after dosing stops.^13,14^

Existing pharmacokinetic methodologies can address many of these problems, but only when they are deliberately applied. Rich sampling across the early absorption and late terminal phases can help separate absorption from elimination. Intravenous reference data, when ethical and available, can identify true systemic disposition. Formulation comparisons, deconvolution methods, population pharmacokinetic models with transit and depot compartments, and physiologically based pharmacokinetic (PBPK) models can all estimate absorption-limited behavior more rigorously than non-compartmental analysis alone.^6^ For biologics, target-mediated drug disposition models and whole-body PBPK frameworks can represent lymphatic uptake, FcRn recycling, tissue distribution, and placental transfer.^13,14,16^

The limitation is that many obstetric pharmacokinetic studies are not designed with these questions in mind. Sparse sampling, short follow-up, trimester-only sampling windows, and reliance on apparent terminal slopes can obscure absorption-limited behavior. Under flip-flop conditions, a model that fits the observed data well can still attribute the terminal phase to the wrong process if it lacks mechanistic structure or external information. Future studies of long-acting therapeutics in pregnancy should, therefore, pre-specify absorption models, including late post-dose and postpartum sampling, evaluate sensitivity to K_a_ and K_e_ assumptions, and report whether terminal half-life is interpreted as elimination-limited or absorption-limited.

## A Revised Framework for Obstetric Pharmacology

Flip-flop pharmacokinetics challenges a common but often implicit assumption in clinical pharmacology, namely that prolonged plasma concentration primarily reflects slow drug elimination. In many modern therapeutic systems, especially long-acting and tissue-targeted agents, the more important question is whether the body is eliminating the drug slowly. Pregnancy makes this distinction more important because maternal physiology changes continuously rather than remaining static.^1–3,8^ A single pharmacokinetic parameter estimated at one gestational age window does not adequately represent absorption, distribution, elimination, and postpartum recovery.

This framework does not replace established pregnancy pharmacology. It complements it. Clearance, volume of distribution, protein binding, metabolism, placental transport, and pharmacodynamics remain essential. The added point is that modern therapeutics increasingly make absorption a programmable, engineered, and clinically decisive process.^6^ Obstetric pharmacology must, therefore, evolve from asking only whether pregnancy increases clearance or lowers exposure to asking which pharmacokinetic process governs exposure at each stage of gestation. Recognition of these principles has major implications for long-acting HIV therapeutics, biologics, fetal therapeutics, immunomodulators, and next-generation precision medicines increasingly used during pregnancy.

A pregnancy-adapted flip-flop framework would classify therapeutics according to route, formulation, expected absorption duration, tissue-depot dependence, lymphatic contribution, maternal clearance pathway, placental transfer mechanism, and postpartum reversal. This would allow clinicians and regulators to distinguish immediate clinical questions from mechanistic uncertainty. For example, long-acting agents may require monitoring for subtherapeutic tail exposure even if peak concentrations are adequate; a biologic may require assessment of fetal exposure even if maternal concentrations decline slowly; and an extended-release oral drug may require formulation-specific evaluation rather than extrapolation from an immediate-release product.

## Future Directions

Several priorities follow from this framework. First, pregnancy pharmacokinetic studies involving depot formulations, subcutaneous therapeutics, biologics, and long-acting injectables should prospectively evaluate whether absorption or elimination governs the terminal phase. Second, model-informed drug development^7^ should incorporate gestational changes in regional perfusion, depot dissolution, lymphatic uptake, maternal metabolism, FcRn dynamics, placental transfer, adipose distribution, extracellular fluid, and postpartum physiology.^2^ Third, regulatory assessment of long-acting therapeutics used by reproductive-aged populations should include pregnancy-specific “tail-phase” characterization when fetal exposure, resistance, toxicity, or prolonged subtherapeutic exposure is clinically relevant.

An additional frontier involves fetal therapeutics and transplacental drug delivery. As biologics, nanoparticle systems, gene-editing platforms, and targeted immunotherapies increasingly interact with placental transport pathways, prolonged maternal tissue release, and delayed fetal exposure will become dominant determinants of fetal pharmacology. Under these conditions, the placenta itself will increasingly function as both a pharmacokinetic barrier and a secondary drug reservoir.

Future drug development programs involving reproductive-aged populations should anticipate that pregnancy will fundamentally alter not only the magnitude of drug exposure, but also the dominant pharmacokinetic process governing exposure itself. This represents a conceptual shift for obstetric clinical pharmacology. Pregnancy should no longer be viewed merely as a modifier of clearance, but as a dynamic systems-level biologic state capable of reorganizing the relative hierarchy of absorption, distribution, and elimination processes.

Finally, the field of obstetric pharmacology needs better empirical data. At present, many pregnancy-specific flip-flop concerns are biologically plausible but not definitively established. That uncertainty should not lead to an overstatement, but it should also not lead to neglect. The correct response is prospective study design, transparent modeling, adequate sampling, and explicit reporting of the assumptions used to distinguish absorption-limited from elimination-limited exposure.

## Conclusion

Flip-flop pharmacokinetics is not new, and it is not unique to pregnancy. Its importance in obstetric pharmacology lies in the collision between a dynamic gestational physiology and a therapeutic era increasingly defined by long-acting, depot-based, subcutaneous, and biologic platforms. In these systems, prolonged drug exposure may not reflect slow elimination. It may reflect slow entry into the systemic circulation. This central insight is simple but consequential.

Recognizing this distinction can improve the interpretation of pharmacokinetic data, sampling design, model-informed-dose optimization, discontinuation planning, fetal exposure assessment, and regulatory evaluation. Pregnancy should, therefore, be viewed not only as a modifier of clearance, but as a dynamic biologic state capable of reorganizing the relative hierarchy of absorption, distribution, and elimination processes. For the next generation of obstetric therapeutics, that distinction is essential.

## Figures and Tables

**Table 1.** When to Suspect Flip-Flop Pharmacokinetics in Pregnancy

Clinical or Pharmacokinetic Signal	Why It Matters in Pregnancy
Long apparent half-life after non-IV dosing	May reflect slow absorption rather than slow elimination
Prolonged “*tail*” after stopping therapy	Suggests continued drug release from depot or tissue compartmen
Delayed T_max_ or flattened concentration-time curve	Supports absorption-limited systemic entry
Lower peak but sustained concentrations	Common with depot, extended-release, or subcutaneous products
Discordance between expected clearance and observed half-life	May indicate that terminal slope is governed by absorption
Greater trimester-to-trimester variability	Pregnancy physiology may change depot release, tissue perfusion, oi gastrointestinal absorption.

**Table 2.** Therapeutic Settings in Which Flip-Flop Pharmacokinetics Should be Considered During Pregnancy

Therapeutic Setting	Reason Flip-Flop Would Matter	Pregnancy-Specific Concern	Practical Implication
Long-acting injectable antiretrovirals	Sustained intramuscular depot release drives prolonged tail-phase exposure	Gestational and postpartum changes can alter depot release or efficacy thresholds	Plan late sampling, postpartum follow-up, and resistance-aware discontinuation strategies
Depot hormonal therapies	Systemic exposure depends on slow release from tissue reservoirs	Exposure variability can be influenced by adipose distribution and injection-site physiology	Interpret apparent half-life as formulation-dependent unless elimination is separately characterized
Subcutaneous biologies and monoclonal antibodies	Absorption can depend on lymphatic uptake and FcRn-mediated processes	Placental FcRn transfer and immune adaptation can change maternal and fetal exposure	Use biologic-specific PBPK or population models with maternal, placental, and fetal compartments
Extended-release oral formulations	Dissolution or intestinal absorption can become slower than elimination	Gestational gastrointestinal changes can prolong or destabilize absorption	Avoid assuming immediate-release behavior in pregnancy studies
Implants, nanoparticles, and engineered delivery systems	Release kinetics are intentionally prolonged and formulation-controlled	Pregnancy may alter tissue distribution, sequestration, and postpartum release	Require pregnancy-specific tail-phase and fetal exposure characterization

## Data Availability

Data sharing is not applicable to this article, as no new or de novo datasets were generated or analyzed. This work is a scholarly commentary based on the synthesis, interpretation, and contextualization of previously published literature and publicly available information.
